# Oxidative Stress and the Pathophysiology and Symptom Profile of Schizophrenia Spectrum Disorders

**DOI:** 10.3389/fpsyt.2021.703452

**Published:** 2021-07-22

**Authors:** Alex J. Murray, Jack C. Rogers, Mohammad Zia Ul Haq Katshu, Peter F. Liddle, Rachel Upthegrove

**Affiliations:** ^1^Institute for Mental Health, University of Birmingham, Birmingham, United Kingdom; ^2^Institute of Mental Health, Division of Mental Health and Neurosciences University of Nottingham, Nottingham, United Kingdom; ^3^Nottinghamshire Healthcare National Health Service Foundation Trust, Nottingham, United Kingdom; ^4^Early Intervention Service, Birmingham Women's and Children's National Health Service Foundation Trust, Centre for Human Brain Health, University of Birmingham, Birmingham, United Kingdom

**Keywords:** schizophrenia, oxidative stress, psychosis symptoms, antio×idants, dopamine, glutamate, inflammation

## Abstract

Schizophrenia is associated with increased levels of oxidative stress, as reflected by an increase in the concentrations of damaging reactive species and a reduction in anti-oxidant defences to combat them. Evidence has suggested that whilst not the likely primary cause of schizophrenia, increased oxidative stress may contribute to declining course and poor outcomes associated with schizophrenia. Here we discuss how oxidative stress may be implicated in the aetiology of schizophrenia and examine how current understanding relates associations with symptoms, potentially *via* lipid peroxidation induced neuronal damage. We argue that oxidative stress may be a good target for future pharmacotherapy in schizophrenia and suggest a multi-step model of illness progression with oxidative stress involved at each stage.

## Introduction

Schizophrenia is a severe and debilitating mental disorder that has an estimated life-time prevalence worldwide of 0.75% (1). Long term outcomes of this disorder are often poor, and those diagnosed with schizophrenia are up to three times more likely to die early than the general population in spite of treatment (2). Schizophrenia is characterised by positive, negative, and disorganisation symptoms. Positive symptoms include hallucinations (perceptual experiences in the absence of corresponding stimuli, for example: hearing voices or seeing things that are not there) and delusions (unshakeable beliefs arising internally, e.g., a delusion of grandeur occurs when a person believes themselves to be superior to others with no evidence for this). Negative symptoms include loss of motivation, apathy and social withdrawal. Disorganisation includes disordered form of thought and inappropriate affect. Negative and disorganisation symptoms occur alongside impaired cognitive function and deterioration in both social and occupational functioning (3, 4). Schizophrenia has an age of onset of between 18 and 25 years in men and 25–35 years in women (5), with a prodromal phase that can be detected up to 30 months before onset (6).

Oxidative stress is caused by an excess of free radicals generated by cellular metabolic stress and an impaired antioxidant defence system, and is known to cause membrane dysfunction implicated in the pathophysiology of schizophrenia (7). However, our understanding of schizophrenia and the involvement of oxidative stress is constantly evolving in the wake of new neurobiological methodologies, such as magnetic resonance spectroscopy, used to assess *in vivo* metabolites within the brain. The advancement of new technologies and increased understanding will enable us to develop novel treatments to target clinical symptoms and identify preventative mechanisms to halt transition to schizophrenia in individuals at high risk for mental health disorders.

Despite this, the mechanisms of different psychotic disorders, including schizophrenia, are not fully established, with evidence supporting a number of theories including neuronal maldevelopment (8), hyperactive dopamine transmission (9), hypoactive glutamatergic signalling (10) and immune dysfunction (11), including microglial dysfunction (12), and overproduction of inflammatory cytokines via innate immune cells (13). However, one commonality between many of these theories is altered function of the neuronal membrane, along which is a litany of neurotransmitter receptors and ion channels. The neuronal membrane is the functional site of drug effects and signal transduction (14) and, furthermore, represents a point where both genetic and environmental factors related to the aetiology of schizophrenia may interact (15).

This review will explore the mechanisms by which oxidative stress may affect the brain and how this may be related to the symptom profile of schizophrenia. Initially we will provide a brief description of oxidative stress, covering free radicals and exploring endogenous antioxidant defences. We will then examine the literature on impaired antioxidant defence mechanisms in schizophrenia, including the methods by which this is assessed, before evaluating how these mechanisms may relate to the symptom profile of schizophrenia, including positive, negative and disorganised symptom severity. The studies were found via PubMed and Google Scholar searches using combinations of key words “schizophrenia,” “oxidative stress,” “antioxidant defence,” “magnetic resonance spectroscopy,” “dopamine,” “glutamate,” “mTOR” “inflammation,” “dysconnectivity,” and “symptoms.” The searches yielded original research, meta-analyses and review articles that were peer reviewed and in English.

## Oxidative Stress

Oxidative stress is defined as an imbalance between the production and subsequent build-up of reactive species, or free radicals, and the body's inability to detoxify these reactive products. This in turn can lead to molecular and cellular damage (16). There are two types of reactive species: reactive oxygen species (ROS), such as superoxide (${\text{O}}_{2}^{•-}$) or hydrogen peroxide (H_2_O_2_), and reactive nitrogen species (RNS), such as the nitroxyl anion (NO^−^) and various nitrogen oxides (NO_2_, N_2_O_4_, etc.) (17). Reactive oxygen species are generated as by-products of mitochondrial production of adenosine triphosphate (ATP), a crucial molecule for cellular actions (18). The electron transport chain employed in this production consumes roughly 90% of all oxygen absorbed by the cells (19) with an estimated 0.1–0.5% of this oxygen being converted into superoxide radicals (20).

Free radicals, such as the superoxide radical, are known to have some beneficial physiological effects; e.g., they can aid the body's innate immune system and provide a key line of defence against pathogens (21). In a healthy state the level of free radicals is controlled to maintain a balance between oxidation and reduction in tissues (22). However, when production of these species increases, such as when the body is in a high stress condition or disease state, they begin to negatively affect important structures within cells, such as lipids, proteins and nucleic acids (23). One example of this is when the hydroxyl radical and peroxynitrite are in excess, they can cause lipid peroxidation which in turn damages cell membranes and lipoproteins. This can lead to the formation of malondialdehyde and conjugated diene, both of which are known to have toxic and mutagenic properties (24). Furthermore, neurons within the central nervous system are at risk of damage from reactive species (25). The brain has high levels of oxygen consumption, around 20% of total basal oxygen consumption and an increased rate of oxidative metabolism. These factors, combined with lower levels of protective antioxidant enzymes and a high proportion of easily oxidised membrane polyunsaturated fatty acids (PUFAs), when compared with the rest of the body, lead to a much greater risk for the negative effects of oxidative stress (26).

To combat excessive accumulation of ROS and RNS there is a complex set of endogenous antioxidant defences, both enzymatic and non-enzymatic. Antioxidant enzymes such as Superoxide Dismutase (SOD), Catalase (CAT), and Glutathione Peroxidase (GPx) help to block the initiation of reactive species chain reactions and form the first line of antioxidant defence (25). These enzymes act in conjunction to inactivate the superoxide radical. ${\text{O}}_{2}^{•-}$ is transferred into H_2_O_2_ via the addition of an electron in a reaction catalysed by SOD. The hydrogen peroxide produced by this reaction is then decomposed into harmless water and oxygen by CAT and GPx (27). As each of these enzymes is critical in different stages of free radical metabolism, change in activity of one without compensation by the others could leave cellular membranes vulnerable to damage (28).

The second line of defence comes from non-enzymatic antioxidant components such as glutathione (GSH), metal binding proteins (MBPs) and uric acid (UA) which rapidly inactivate reactive species and thereby prevent the propagation of chain reactions (17). These non-enzymatic antioxidants work in a number of ways to help neutralise excess free radicals. MBPs inhibit the formation of new reactive species by binding metals such as iron and copper (29), whereas GSH is a free radical scavenger; it scavenges reactive species and inactivates them. During the reaction GSH is oxidised into glutathione disulphide GSSG, which can then be reduced back into GSH (30). Additionally, dietary antioxidants such as vitamin E, vitamin C and carotenoids can affect the activity of endogenous antioxidants, with vitamin C helping to support the regeneration of GSSG back into GSH (31).

To summarise, the human body has to maintain a delicate balance of forming enough reactive species to perform useful physiological roles, whilst breaking down the excess to prevent unnecessary cellular damage. As such oxidative stress is thought to play a key role in many physical disorders such as cardiovascular disease and diabetes (32, 33), as well as a number of mental disorders such as depression and schizophrenia (34, 35).

## Oxidative Stress and Schizophrenia

Increased levels of reactive species and decreased levels of antioxidant defences are seen to cause oxidative damage to a number of cellular structures. Many studies have now shown that oxidative damage is present in schizophrenia (14, 36, 37). Although this may not be the primary cause of schizophrenia, growing evidence has suggested that it may contribute to the declining course and poor outcome in schizophrenia (38).

Examining oxidative stress within the brain is particularly difficult because, until recently, there was no way to assess metabolite concentrations in living human tissue. As such, a variety of methods have previously been employed to assess oxidative stress within schizophrenia. A large number of studies have assessed peripheral biomarkers of oxidative stress such as antioxidant levels. Total antioxidant and glutathione levels have been shown to be lower within the plasma of non-medicated, medicated, first-episode and chronic schizophrenia patients (39–42). In addition to this, increased levels of reactive oxygen species have been found in the periphery of schizophrenia patients (43, 44), in conjunction with reduced levels of SOD and GPx (45). Furthermore, redox regulatory findings have been shown to be influenced by illness phase e.g., stable or acute schizophrenia (46). Post-mortem studies also report reduced glutathione levels in the brains of schizophrenic patients, specifically within the prefrontal cortex and the caudate (47, 48), with abnormal protein expression in the anterior cingulate cortex (ACC) a result of increased oxidative stress (49).

Results from *in vivo* MRS studies of glutamate/glutamine concentrations in schizophrenia, whilst inconsistent, have highlighted that sub-grouping patients on “residual schizophrenia” (long-term negative symptoms/impairments) revealed reduced, highly correlated, GSH, glutamate and glutamine concentrations in the ACC (50, 51). Lower grey matter volume (GMV) in medial frontal and ACC in ultra-high-risk individuals for schizophrenia also predicted poorer long-term functional outcome at follow-up (~9 years later), irrespective of transition to schizophrenia or persistence of at-risk mental state (52).

It has been suggested that, in schizophrenia, redox dysregulation and subsequent oxidative stress may be limited to a specific subgroup representing ~1 third of patients (46, 53–55). This subgroup is characterised by very low levels of polyunsaturated fatty acids (PUFAs) within red blood cells during the acute phase of illness (53), when PUFAs were bimodally distributed, as well as deleterious effects of eicosapentanoate (EPA) or vitamin E and C on mental functioning (54, 55). During a stable phase, PUFA was no longer bimodally distributed, but high 2-amino butyrate in the low PUFA group indicated persistent redox dysregulation (46).

### Genetic Studies

Genome wide association studies have shown an association between gene polymorphisms for oxidative stress and schizophrenia (56). Genetic variations have been found in the strands of DNA that code for the rate limiting enzyme, glutathione cysteine ligase and glutathione-S-transferases, involved in the synthesis of glutathione (57). A high-risk genotype for the glutathione cysteine ligase catalytic unit has been linked to impaired capacity to synthesise GSH under conditions of oxidative stress, as well as a reduction in medial prefrontal GSH levels (58, 59). Genome wide association studies have also identified a “psychiatric susceptibility gene” cacna1c as one of the strongest genetic risk factors for the development of affective disorders (60). This gene has recently been linked to mitochondrial function and subsequent oxidative stress (61), suggesting it may play a key role in the aberrant generation of damaging reactive oxygen species seen in schizophrenia.

### Animal Models

Higher levels of reactive species within mitochondria have been found in the brains of ketamine-induced rat models of schizophrenia compared to wild-type controls (62). Another rodent model of schizophrenia is the N-methyl-D-aspartate-antagonist MK-801-induced model, and this too has been found to have increased levels of oxidative stress in the prefrontal cortex (63). Glutathione depletion after the administration of 2-cyclohexen-1-one, a chemical which enhances the rapid degradation of GSH has also resulted in schizophrenia-like behaviour in rodents (64). Knockout mice that lack a subunit of glutamate cysteine ligase have demonstrated a significant reduction of GSH in the anterior cingulate cortex (65), which has resulted in schizophrenia-like behaviour, including hyperlocomotion and altered social behaviour (66). Furthermore, these knockout mice demonstrate neuronal changes within the hippocampus similar to those seen in schizophrenia, specifically decreasing the numbers of parvalbumin interneurons (67).

Evidence of redox dysregulation is seen in other common neurodevelopmental animal models of schizophrenia. For example, the social isolation rearing model explores the effects of environmental insults via social deprivation on the developing brain after birth, with animals developing specific behaviours and neurobiology akin to schizophrenia (68, 69). Within this model increases in SOD activity are seen in conjunction with higher levels of lipid peroxidation in the prefrontal cortex (70). Furthermore, mitochondrial dysfunction is also noted, with increased striatal and decreased frontal cortex ATP (71). Inflammatory mouse models have also shown evidence of increased oxidative stress with prenatal exposure to the bacterial endotoxin lipopolysaccharide shown to decrease levels of GSH in the hippocampus (72). Several studies have demonstrated a significant increase in lipid peroxidation when inflammation is induced postnatally (73–75).

### Clinical Trials

Several studies report that antioxidant treatment has had positive effects on schizophrenia, although the evidence is inconsistent. Treatment with the antioxidant N-acetylcysteine has been of great interest in recent years, with studies finding that it ameliorated depressive symptoms (76–78) and may reverse oxidative stress induced by mitochondrial dysfunction (79). A recent meta-analysis by (80) concluded that n-acetylcysteine led to improvement in negative symptom score, total symptom score and working memory. Sulforaphane, another antioxidant, has been shown to have neuroprotective properties (81) and may reduce the risk of transition to schizophrenia from an at-risk state (82). Sedlak et al. (83) demonstrated that treatment with sulforaphane increased the levels of available GSH in the brains of healthy controls after 7 days. Results from animal trials have suggested that dietary intake of the sulforaphane precursor glucoraphanin prevented cognitive deficits in adult offspring after maternal immune activation (84). Additionally, a small study of seven human patients with schizophrenia found a significant improvement in a test of working memory after an 8-weeks treatment with sulforaphane, although the sample size may have been too small to detect any other improvements (85). Due to the positive results seen from these studies clinical trials are now underway to assess the efficacy of sulforaphane in clinical subjects.

Further research into dietary antioxidants such as vitamins and Omega-3 PUFAs have been of interest in recent years (86). Vitamin C and E have been reported to improve patient symptoms (87), however, more recent studies have shown that in high doses, these vitamins may act as pro-oxidants and can increase the levels of oxidative stress, although when combined with ethyl-EPA, vitamin E and C in these high doses was not deleterious (54, 88). Omega-3-PUFAs are shown to be reduced in schizophrenia (38, 89) and act as an essential building block of eicosanoids which act to regulate inflammation and oxidative stress (90). Studies assessing Omega-3 PUFA supplementation have yielded mixed results with some demonstrating a reduction in symptom severity (91, 92) and others have found no additional benefit compared to placebo (93–95). It has been suggested that perhaps the reason for these mixed results is due to illness phase and those with chronic schizophrenia may have progressed too far for supplementation to have a beneficial effect (86). Indeed one meta-analysis suggested that Omega-3 PUFA supplementation was most effective in earlier phases of illness and reduces the conversion from high risk to first episode (96). Furthermore, it has been suggested not only dietary supplementations but elimination of substances which are toxic or not tolerated by some patients may have a beneficial effect in schizophrenia treatment. For example a recent study showed that the removal of gluten from a patients diet was associated with a reduction in negative symptom severity (97).

It should be noted that there is some contention as to whether peripheral biomarkers can reflect the status of the CNS (25). Traces of oxidative damage may arise from a variety of areas within the body and as such peripheral indicators of oxidative stress may not reflect the conditions within the brain (59, 98). Whilst some studies have shown peripheral antioxidant capacity is consistent with the central nervous system (99), peripheral status is simply indirect evidence. As a result of this, the next step is to assess oxidative status *in vivo*. The primary method by which this can occur is the use of magnetic resonance spectroscopy (MRS).

### Magnetic Resonance Spectroscopy

MRS is a relatively new tool that is used in conjunction with MRI to non-invasively measure the concentration of metabolites within living tissue (100). It can be used to assess antioxidant concentrations in the brain (101). Similar to magnetic resonance imaging (MRI), MRS acquires a signal from hydrogen protons. However, while MRI acquires signal primarily from protons within water and fat, due to their high concentration within the brain, MRS acquires its signal from other molecules, such as GSH. By examining the difference in resonance frequency of hydrogen nuclei in difference chemical environments, MRS can distinguish between hydrogen nuclei in different molecules. Hydrogen protons seen in fat and water are approximately one thousand times more abundant than those detected in molecules by MRS (102) and thus MRS employs a method to suppress the water proton signal. Since the molecules of interest within MRS studies are much less abundant than water, larger voxels of acquisition are required, typically 2 × 2 × 2 cm^3^ (103). Larger voxels help to improve the signal to noise ratio which is often poor in MRS studies (104). However, with a larger voxel comes difficulties in voxel composition, a 2 cm^3^ voxel makes it incredibly difficult to get a “pure” white matter location and virtually impossible to obtain a “pure” grey matter placement (104).

In light of the evidence for extensive structural and functional brain abnormality in schizophrenia (105), the question of optimum placement of an MRS voxel for identification of relevant abnormalities in antioxidant concentrations in schizophrenia remains unanswered (106). Nonetheless, meta-analyses of convergent GMV loss across diverse psychiatric diagnostic groups (107), post-mortem studies of tissue from patients and also the evidence from relevant animal models of schizophrenia reviewed above suggest that the prefrontal cortex, anterior cingulate and medial temporal lobe including hippocampus, are candidate regions of interest. Furthermore, in mice the highest concentrations of GSH are in the cortex followed by the cerebellum, hippocampus and striatum (108) suggesting differential sensitivity of different brain regions to damage from oxidative stress. There is also the issue of grey matter or white placement, with GSH concentrations shown to be 30% higher in white matter than grey matter (109), however grey matter is seen to be more vulnerable to oxidative stress (110). Hence, no ‘gold standard' approach has resulted in studies often choosing different areas of the brain to assess antioxidant concentrations resulting in varied findings.

## Interaction of Oxidative Stress and Current Schizophrenia Hypotheses

### The Dopamine Hypothesis

The dopamine hypothesis is the most well-known in schizophrenia and has dominated the literature for many years. This theory was proposed after it was discovered that the drug chlorpromazine had antipsychotic properties (111), further to this, Carlsson and Waldeck (112) discovered that dopamine was a neurotransmitter. It was subsequently proposed that the therapeutic effects were the result of selective blockade of dopamine D2 receptors (113). To this day, all antipsychotic drugs act on dopaminergic receptors in the brain (9). It is proposed that D2 receptor neurotransmission is hyperactive within subcortical and limbic brain regions. This hyperactivity is thought to contribute toward positive symptoms in schizophrenia. Alongside D2 hyperactivity, it is also thought that D1 receptor hypoactivity can contribute toward the negative and cognitive symptoms seen in this disorder (114). Post mortem studies have found an increased density of D2 receptors in the brains of schizophrenia patients (115). Upregulation of D2 receptors within the caudate nucleus is also reported to correlate with cognitive dysfunction (116). Additionally, indirect dopamine agonists, such as amphetamine and cocaine, have been shown to induce positive symptoms in the general population (117) with schizophrenia patients displaying an increased sensitivity to the dopamine-releasing effects of these drugs (118–120).

However, antipsychotic drugs tend to alleviate positive symptoms more than negative symptoms (121), with some studies showing that they may worsen negative symptoms in patients (122) and even induce them in healthy controls (123). It has been suggested that hypoactivity of the dopamine pathway is a mediator of negative symptoms in schizophrenia, indicating that reduced dopamine activity may be the end difficulty rather than dopamine overactivity (124).

Metabolism of dopamine has been suggested to be a prominent producer of reactive oxygen species in the brain (26). Oxidation of dopamine (both enzymatic and non-enzymatic) results in the generation of H_2_O_2_ which when in the presence of iron or oxygen can form the more active hydroxyl radical (^.^OH) (125). Additionally, the oxidation of dopamine can form dopamine quinones; these could then react with the sulfhydryl groups of glutathione, thus reducing the levels of GSH and increasing the levels of ROS (126). One study found that dopamine alone caused a 40% reduction in GSH levels within cortical neurons (127).

### The Glutamate Hypothesis

Initially proposed in 1980 after glutamate levels were seen to be lower in the cerebrospinal fluid of schizophrenia patients (128), the glutamate hypothesis postulates that the negative symptoms seen in schizophrenia are in part linked to dysfunctional glutamatergic signalling, mediated by NMDA receptors on GABAergic interneurons (10). Similar to the dopamine hypothesis, initial support for this theory came from studies into mind altering drugs. NMDA receptor antagonists, such as ketamine, are seen to induce psychosis in healthy controls (129), with the induced psychosis caused by NMDA receptor antagonists resembling schizophrenia symptoms more closely than those that act on the dopaminergic system (130).

A number of genes have been seen to influence the function of glutamate receptors (131, 132). Reports from genome wide association studies have found that out of 108 loci related to schizophrenia risk, six involve genes implicated in brain glutamate function, with many more thought to affect glutamate function indirectly (133). Bustillo et al. (134) found that polymorphisms in the glutamate-related genes CLCN_3_ GRM_3_ and SLC_3_8A_7_ were directly correlated with combined glutamate and glutamine signal in the grey matter of younger schizophrenia patients (<36 years). Post-mortem studies have found a reduction in NMDA receptors in the brain tissue of schizophrenia patients (135). Moreover, brain imaging studies have demonstrated reduced binding of NMDA receptors in the hippocampus of schizophrenia patients (136).

**Figure 1:**
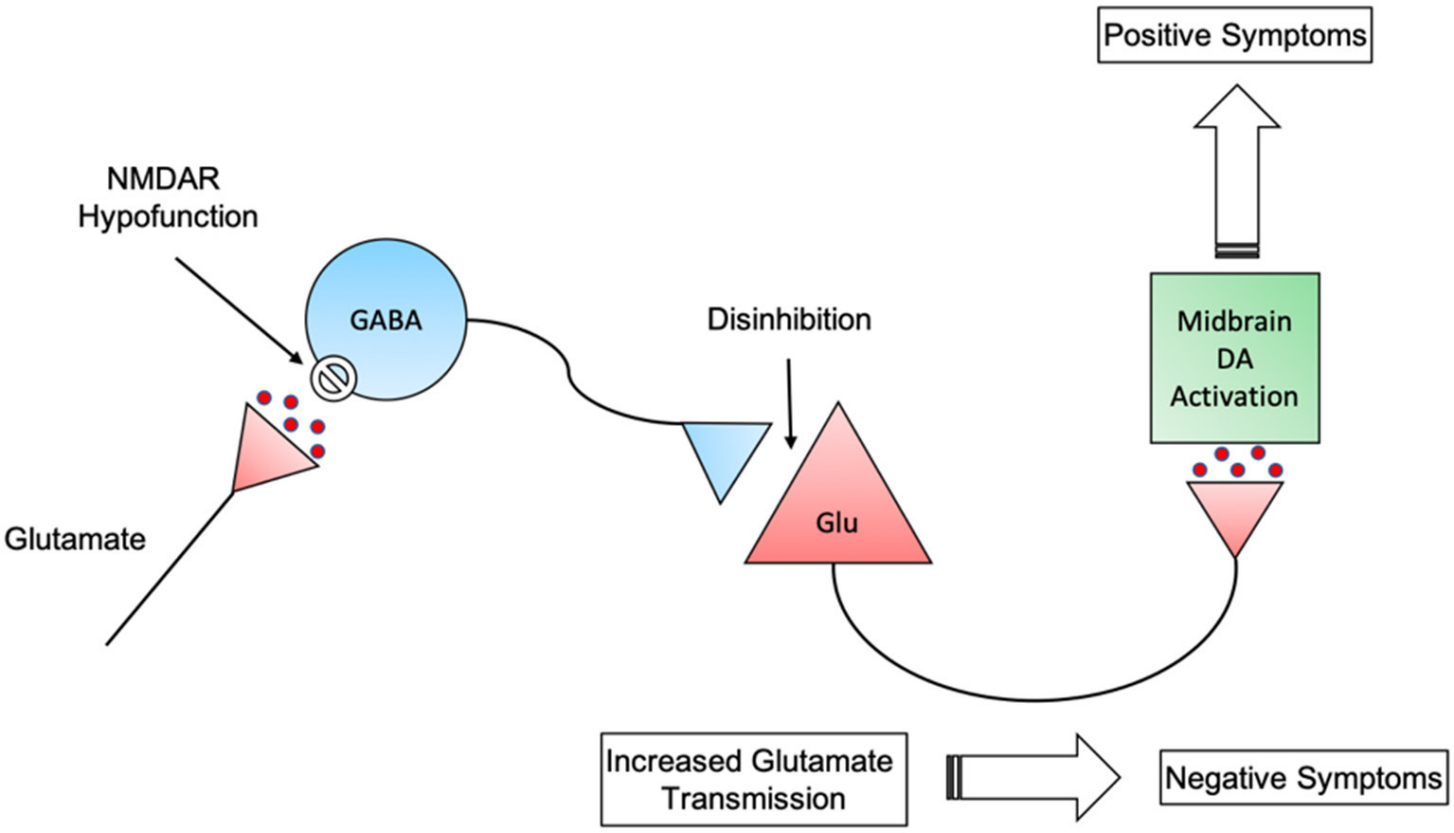
Interactions between GABAergic disinhibition of glutamatergic neurons and subsequent stimulation of midbrain dopaminergic neurons. NMDAR, N-methyl-D-aspartate receptor; GABA, Gamma-Aminobutyric acid; Glu, Glutamate; DA, Dopamine.

Although the glutamate hypothesis may be closer to the root cause of schizophrenia, it does not rule out the dopamine hypothesis. Recent circuit-based models have implicated both glutamatergic and dopaminergic neurotransmission in the pathogenesis of schizophrenia (137). Dopamine neurons are regulated by glutamatergic inputs to the midbrain dopamine nuclei. As such, dopamine function may be secondary to aberrant glutamate functioning. NMDA receptor dysfunction on GABAergic interneurons leads to disinhibited glutamate transmission which could result in the appearance of negative symptoms. Furthermore, these glutamate neurones project into the midbrain and activate the dopamine pathways that are key to positive symptom appearance (Figure 1) (138).

Results from animal studies show that GSH and glutamate are closely related (139). Glutathione synthesis is directly related to glutamate uptake in microglia and the subsequent release of glutamate metabolites (140); additionally, glutathione depletion as a result of oxidative stress is strongly related to microglial glutamate release (141). Activation of glutamatergic pathways can trigger the generation of free radicals while reducing endogenous protection against free radical damage (142). Furthermore, free radicals can trigger the release of glutamate into the synaptic cleft while blocking its reuptake (143).

GSH and NMDA receptor activity are closely linked; an increase in glutathione levels is shown to raise NMDA receptor responsiveness, whereas its depletion has resulted in NMDA receptor hypofunction (144, 145). Hypofunction of these receptors can result in increased free radical production and subsequent oxidative damage (146). One more recent study has shown that synaptic NMDA receptor activity is intrinsically linked to GSH production, with an increase in synaptic activity triggering a subsequent growth in GSH production and utilisation (147). Taken together these studies demonstrate the close link between the glutamatergic system, NMDA receptor hypofunction and GSH.

### mTOR Pathways

More recently a new hypothesis for schizophrenia aetiology has arisen; the mammalian target of rapamycin (mTOR) hypothesis (148). The mammalian target of rapamycin (mTOR) acts as a central regulator of cell metabolism, growth and proliferation via the integration of both intra and extracellular signals (149). Misregulation of mTOR via upstream proteins phospotidylinositol 3-phosphate kinase (PI3K) and protein kinase B (PKB) is thought to contribute to schizophrenia (150, 151). Inhibition of either PI3K/PKB or mTOR leads to inhibited of neuronal growth and thus might contribute to the aberrant synaptic architecture seen in schizophrenia (152). As a result of this inhibited neuronal growth there is a reduction in dendritic branching and subsequent synaptic formation (153). This may result in the development of negative symptoms due to the lack of neuronal connexions (148). Overstimulation of the mTOR system in specific brain areas has been linked with cognitive deficits seen in schizophrenia (154). While inhibition of this pathway causes reduced dendritic branching, overstimulation is thought to increase the number of synaptic connexions, thus leading to the generation of positive symptoms (155). The mTOR pathway has been shown to have connexions to both serotonin and glutamatergic pathways in the brain, through interactions with the serotonin receptor 5-HT6 (156) and glutamate receptors mGluR and NMDA (157), thus linking the mTOR pathway to the glutamate hypothesis.

Oxidative stress can also interact with the mTOR pathway resulting in the development of cognitive symptoms within schizophrenia. It has been proposed that cognitive symptoms arise from prefrontal cortex dysconnectivity (158). This dysconnectivity has been related to myelin and oligodendrocyte abnormalities in schizophrenia patients (159). Myelin is produced by mature oligodendrocytes, the precursor of which is particularly susceptible to oxidative stress (160). It has been proposed that reactive oxygen species can inactivate sections of the mTOR pathway leading to reduced myelination and proliferation of the oligodendrocyte precursors and subsequent disruption of connectivity within the prefrontal cortex (161).

### The Immune Hypothesis

Long before the development of modern antipsychotics, infections and inflammation were proposed to be the cause of psychosis. During an influenza pandemic in the late 19th century it was demonstrated that psychiatric conditions could be caused by an infectious agent, in this case the flu, and one specific type of infection can produce a number of different psychiatric syndromes (162). These insights are still valid today (163); however, they have become more generalised, with inflammation thought to play a key role in many psychiatric disorders in the absence of an acute infectious disease (164).

Pro-inflammatory cytokines, astrocytes, microglia and immune cells such as macrophages and T- or B- lymphocytes help to mediate inflammation in the CNS (165). Under normal circumstances these inflammatory mediators play an essential role in combating infection, harmful chemicals and responding to tissue damage (166). However, dysregulation of the inflammatory response, for example via infection, can trigger a cascade which affect central nervous system (CNS) processes and behavioural phenotypes (167). This dysregulation is central to the immune hypothesis of schizophrenia. Inflammation could cause significant CNS changes which result in the appearance of positive, negative, and disorganised symptoms (168).

Genome wide association studies have located key risk genes for schizophrenia within the major histocompatibility complex (MHC) on chromosome 6, a key loci that codes for specific cell surface proteins essential within the immune system (169). Complement component 4 (C4), a gene located within the MHC which affects both synaptic pruning and opsonization of pathogens, is of particular interest. Recent studies have shown that people with schizophrenia overexpress this gene, thus causing a disruption in synaptic pruning and inflammation related damage (170). Furthermore, overexpression of C4 may help to explain the developmentally timed nature of schizophrenia (171). Additional polymorphisms on genes coding for inflammatory cytokines have also been implicated in schizophrenia risk (172).

Further clinical studies have found increased biomarkers of neuroinflammation in schizophrenia patients, including greater levels of circulating inflammatory cytokines such as Interleukin-6 (IL-6), Tumour Necrosis Factor Alpha (TNF-α) and Interferon Gamma (IFN-γ) (35, 173–176). Elevated cytokine levels are seen to arise before the onset of schizophrenia (177) and may even predict later transition from an at risk mental state, for example elevated levels of IL-6 at age 9 are shown to double the risk of a psychotic disorder diagnosis at age 18 (35). Higher levels of C-reactive protein (CRP) at age 15 are also associated with an increased risk of schizophrenia development by age 27 (178).

It has been hypothesised that these inflammatory cytokines may result in the appearance of the schizophrenia phenotype via disturbance of key neurotransmitter systems (179). Evidence has suggested that pro-inflammatory cytokines increase the concentration of kynurenic acid, a naturally occurring NMDA receptor antagonist, triggering hypofunction of the NMDA receptor and thus promoting increased glutamatergic transmission, ultimately resulting in schizophrenia symptoms (180).

One potential cause of the neuroinflammation seen in schizophrenia could be maternal immune activation (MIA) (181). Studies have shown an association between maternal infection in pregnancy and schizophrenia development in offspring (182). Further studies have shown that this is not dependant on the type of infection the mother has, immune activation alone, and the subsequent cytokine release, is enough to significantly increase schizophrenia risk in offspring (183). It has been suggested that 14%-21% of all schizophrenia cases could be prevented by the eradication of maternal influenza (184).

Inflammation and oxidative stress are intrinsically linked. Tissue damage caused by oxidative stress can trigger inflammation and an immune response (185). Furthermore, macrophages and microglia use reactive oxygen species to kill pathogens (186). As such oxidative stress can be seen as both an inducer and a product of inflammation (187). In addition to this, the imbalance between pro and anti-oxidants may play a key role in the maternal immune activation model (72). In mice it has been demonstrated that MIA resulted in the elevation of a number of oxidative stress markers, including glutathione (188). Taken together it appears that inflammation and oxidative stress have a close reciprocal relationship within schizophrenia.

### The Dysconnectivity Hypothesis

One of the more prominent schizophrenia hypotheses today is the dysconnectivity hypothesis. First proposed in 1995 by Friston and Frith, it suggests that schizophrenia symptoms may arise from disrupted brain connectivity. This hypothesis was based on findings that schizophrenia patients demonstrated a reduction in the connectivity between the prefrontal cortex (PFC) and temporal brain regions (189). Since these initial findings a number of imaging studies have investigated this, each with more descriptive and sensitive techniques [including Dynamic Causal Modelling (DCM), Psycho–Physiological Interaction (PPI) and Independent Component Analysis (ICA)] (190).

A number of white matter abnormalities have been seen in both medicated an unmedicated schizophrenia patients, including a disruption in white matter integrity which is correlated with cognitive impairment (191–193). Importantly this disruption in white matter integrity occurs before the onset of frank schizophrenia and worsens as symptoms progress (194–196). Additionally, diffusor tensor imaging (DTI) has revealed widespread decreases in white matter tracts across a number of long-range pathways within schizophrenia, such as frontal-temporal-limbic, and cortico-cerebellar pathways (197, 198).

In addition to these white matter abnormalities, meta-analyses have revealed grey matter loss across a number of brain sites, including cortical, subcortical, cerebellar and limbic, with this loss becoming more pronounced as the disorder progresses (199, 200). Meta-analyses of GMV loss also reveals reduced integrity of anterior insula and dorsal anterior cingulate based neural systems (e.g., the salience network) linked to psychotic disorders and deficits in executive functioning (107). These deficits are largely attributed to cellular deficits rather than neuronal loss, for example reduced dendritic branching and spine density (201). In spite of these widespread deficits in structural integrity, functional connectivity between brain areas is quite variable. For example, within specific frontal and temporal regions there is a deficit in white matter and subsequent functional connectivity, however in some cases an increase in connectivity is seen (202, 203). In addition to this, connectivity patterns may vary based on whether the brain is at rest or performing a task (204, 205). As such it has been proposed that schizophrenia can be characterised by structural brain deficits with irregular functional hypo or hyper-connectivity patterns.

It has been hypothesised that these functional deficits in connectivity are in part due to myelin abnormalities (206, 207). As mentioned previously myelin is produced by oligodendrocytes, interrupting the production of myelin can lead to functional dysconnectivity and the appearance of frank psychotic symptoms (161). It is here where oxidative stress may play a role, the oligodendrocyte precursors (OPC) are susceptible to oxidative stress, redox dysregulation alongside inflammation and glutamatergic hypofunction to impair the development of OPCs to mature oligodendrocytes, thus impacting neuronal myelination (207, 208). In a series of human and rodent studies, glutathione deficit in the prefrontal cortex was linked to impaired OPC proliferation alongside oligodendrocyte and myelin maturation (209). OPCs and oligodendrocytes are known to have up to six times more reactive oxygen species within them, perhaps due to the increased metabolic activity required to produce myelin (210). As such, these cells are constantly in a state of increased oxidative stress, to which they are already susceptible. It has been indicated that a redox change of as little as 15% can influence the pathways that stimulate oligodendrocyte maturation (211). Additionally, oxidative stress can trigger downregulation of gene expression related to myelination (212). As such the myelin abnormalities and subsequent dysconnectivity of specific brain regions observed within schizophrenia may be due to oxidative-stress induced OPC dysfunction. Furthermore, it has been noted that the impaired development of OPCs to mature oligodendrocytes can be reversed by supplementation with the antioxidant NAC (207, 209).

## Oxidative Stress and Symptom Profile

Although oxidative stress has been implicated in the aetiology of schizophrenia many times (213–215), the method by which it may relate to specific symptoms is still unclear (216). It has been demonstrated that increased levels of reactive oxygen species and a dysfunction in antioxidant defences can cause significant damage to neuronal architecture. Impairments in oxidative status have been linked to cognitive decline and behavioural abnormalities (217). As such schizophrenia symptoms may be a result of damage to the neuronal lipid membrane in specific regions or networks, caused by excess reactive oxygen species (218).

Studies have shown that an increase in antioxidant enzyme activity in red blood cells, plasma and cerebrospinal fluid are associated with tardive dyskinesia, negative symptoms and poor premorbid dysfunction (43, 219, 220). GSH depletion in particular has been linked to the negative symptoms of schizophrenia (221). Lower glutathione levels have been correlated with worse Positive and Negative Syndrome Scale (PANSS) scores and worse community functioning (222, 223). Additionally, patients with residual or deficit schizophrenia, two subtypes of schizophrenia with predominantly negative symptoms, exhibited a greater reduction in GSH levels compared to those with stable schizophrenia (51, 224). It is assumed that the substantial negative symptoms seen in schizophrenia are a result of aberrant glutamatergic transmission mediated by NMDA receptor hypofunction (225). As mentioned previously, GSH, the glutamatergic system and NMDA receptor function are closely related. The links between these may suggest why negative symptoms are so strongly correlated with GSH concentration in specific brain regions.

Previous studies have demonstrated that SOD activity is positively associated with both positive and negative symptoms, as well as general psychopathology in chronic schizophrenia patients (226, 227). However, other studies have found no link between overall symptom severity and SOD activity (228). One study even found that SOD activity was inversely associated with positive symptoms (229). A more recent study has found that gender differences play a role in clinical symptoms, with higher SOD activity correlated with negative symptoms in men, but with positive symptoms in women (230). A recent study has notably found that platelet lipid peroxidation is associated with the severity of disorganisation symptoms (216).

A number of reasons have been proposed as to why results are inconsistent, including: antipsychotic medication confound, variable disease severity, number of psychotic episodes and source of test material (blood, plasma, or serum) (231). Additionally, there are a number of additional confounding factors which may influence results such as, smoking (232) and obesity (233). An alternative argument for this may be that each biomarker for oxidative stress does not work independently, and they could interfere with each other (234). As such it may be the case that each individual biomarker may not have satisfactory diagnostic power (235) and it may be more pertinent to combine several markers to improve diagnostic precision in future studies.

## Discussion

Oxidative stress has been heavily implicated in the pathogenesis of schizophrenia. With a number of studies finding increased levels of reactive species and decreased concentrations of antioxidant defences alongside significant levels of oxidative damage in schizophrenia (37, 39, 43). Evidence for this has come from a variety of study designs, including post-mortem, genetic, animal and clinical trials (47, 61, 67, 82). Oxidative stress may be seen within the light of most current biomedical hypotheses of schizophrenia, and may play an important role in unifying schizophrenia hypotheses in future. Additionally, a small but increasing number studies have implicated oxidative stress in relation to specific symptoms in schizophrenia, with negative, disorganised and cognitive symptoms most evident (221, 227). It may be the case that the symptoms more related to neuronal development in schizophrenia are a result of membrane damage via lipid peroxidation.

**Figure 2:**
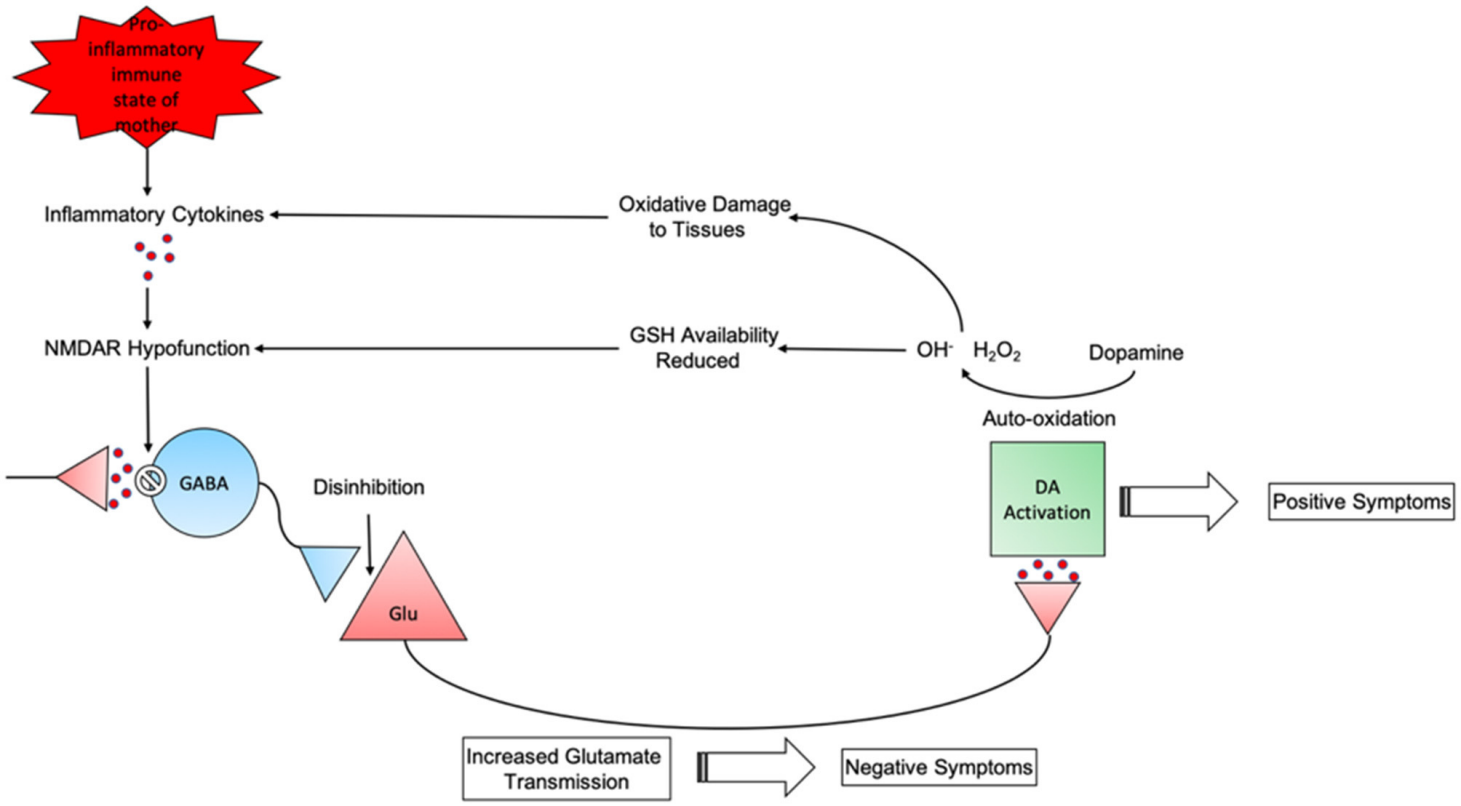
The cyclical nature of schizophrenia progression from maternal pro-inflammatory state to behavioural phenotype.

Taken together these results could present a rough timeline of schizophrenia progression (Figure 2). First, maternal immune activation during pregnancy can cause the release of pro-inflammatory cytokines (181). These cytokines can trigger the overproduction of kynurenic acid, an NMDAR antagonist, thus resulting in NMDAR hypofunction on GABAergic interneurons (180). The hypofunctioning NMDARs result in disinhibition of glutamate neurons which can lead to negative symptoms of schizophrenia (236). These excitatory glutamate neurons project into the midbrain and trigger hyperactivity of dopaminergic pathways which are associated with positive symptoms (237). Treatment with first generation antipsychotics are seen to alleviate these symptoms (238). Catecholamines such as dopamine can auto-oxidate into free radicals (239). Free radicals produced by this will cause tissue damage and increase inflammation (138). In response to increased free radical generation an increase in the antioxidants SOD and GSH are seen to combat this (240). GSH availability is reduced due to the excessive ROS produced by increased dopamine levels (241). As mentioned previously a reduction in available GSH can lead to NMDA receptor hypofunction within inhibitory GABA interneurons (144, 145). Thus, generating a cycle of pyramidal glutamatergic neurotransmission and the generation of diverse symptoms seen in acute schizophrenia (242).

A model such as this provides an opportunity for novel therapeutic interventions in schizophrenia. The biomedical underpinnings of schizophrenia are presented here as a multi-step, cyclical, process that ultimately results in the manifestation of positive symptoms, however such processes are not reflected in current treatments of schizophrenia. Currently antipsychotic medication is prescribed at all stages of the disorder and only effective against positive symptoms. A novel approach would be to use oxidative stress and inflammatory markers as a target for schizophrenia progression and adapting treatment based on the individual, thus moving toward a more personalised approach to schizophrenia treatment (243).

Over the last three decades a large number of clinical trials have been performed and the interest within this research area has been increasing. A 2016 review identified 22 clinical studies of antioxidant treatments in schizophrenia (244), however, authors noted limited evidence for symptom improvements and under-powered study designs. Indeed, varying results from clinical trials are noted in more recent studies, for example, the antioxidant N-acetylcysteine (NAC) has been shown to improve depressive symptoms in patients (78), however, a separate study found that NAC did not improve clinical symptoms or functional outcomes (243). Perhaps these varying results are due to the small sample sizes of the cohorts tested, additionally a recent meta-analysis suggested that longer interventions may be required for antioxidant treatments such as NAC to work, as a significant improvement in symptoms can be seen at 24 weeks or more, but not <8 weeks (80). Oxidative stress presents as a good candidate for schizophrenia intervention, future studies should continue to investigate potential treatments over an extended time course. A broad range of interventions from pharmaceuticals to diet and exercise may have potential to be effective, however stratification based on the patient's biochemical and inflammatory are needed (86, 97, 245). These future studies will not only provide potential new nutraceutical and pharmacological therapies for schizophrenia, they will also allow us to continually improve the knowledge surrounding this complex disorder.

## Author Contributions

AM researched and drafted the article, revised, and edited it. JR critically revised the article, providing support on content, and structure. RU supervised the project, critically revised the article, providing support on content, and structure. PL and MK critically revised the article, providing support on content and structure. All authors contributed to the article and approved the submitted version.

## Conflict of Interest

The authors declare that the research was conducted in the absence of any commercial or financial relationships that could be construed as a potential conflict of interest.
